# LncRNA HOTTIP promotes LPS‐induced lung epithelial cell injury by recruiting DNMT1 to epigenetically regulate SP‐C

**DOI:** 10.1002/ccs3.12020

**Published:** 2024-02-27

**Authors:** Shuang Li, Shuangjia Li, Zhanqun Gao, Yang Liu

**Affiliations:** ^1^ Department of Emergency The First Affiliated Hospital of Jiamusi University Jiamusi China

**Keywords:** acute lung injury, DNMT1, HOTTIP, lncRNA, SP‐C

## Abstract

The objective of this study was to elucidate the involvement of the long noncoding RNA (lncRNA) HOTTIP in acute lung injury and understand the underlying mechanisms. Relevant expression of mRNAs and proteins were assessed by qRT‐PCR and western blot assays. Cell viability was determined by employing the CCK‐8 assay, and apoptosis was quantified through TUNEL staining. The concentration of inflammatory factors was measured by ELISA. The degree of DNA methylation was quantified through MSP assay. The interaction between HOTTIP and DNA methyltransferase 1 (DNMT1) was examined by RIP assay. LPS upregulated HOTTIP, whereas downregulated SP‐C level in AEC II cells. HOTTIP knockdown inhibited LPS‐induced apoptosis and the secretion of inflammatory cytokines (TNF‐*α*, IL‐1*β* and IL‐6) in AEC II cells. Mechanistically, HOTTIP recruited DNMT1 to the SP‐C promoter, thereby facilitating DNA methylation of SP‐C and suppressing its expression. Additionally, inhibitory of SP‐C reversed the effects of HOTTIP or DNMT1 knockdown on apoptosis and inflammation in AEC II cells induced by LPS. HOTTIP recruited DNMT1 to epigenetically inhibit SP‐C expression, leading to the promotion of lung epithelial cell injury caused by LPS, suggesting that targeting HOTTIP may be an effective strategy for the therapy of lung epithelial cell injury.

## Graphical abstract

HOTTIP facilitated apoptosis and inflammation of LPS‐induced lung epithelial cells. Mechanically, HOTTIP recruited DNMT1 to promote the DNA methylation of SP‐C, thereby inhibiting the expression of SP‐C.

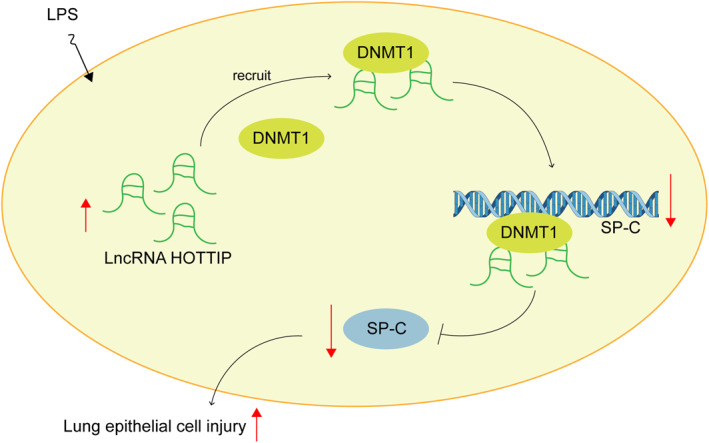

## 1 INTRODUCTION

Acute lung injury (ALI)^1^ is a severe and potentially fatal condition associated with significant morbidity and mortality rates,^2^ which can be caused by various factors, including pneumonia, sepsis, severe trauma, and acute pancreatitis.^3^ If left untreated, ALI can advance into acute respiratory distress syndrome, which represents a more critical and severe manifestation of lung injury.^4^ Nevertheless, the precise pathogenesis of ALI remains unclear. Lipopolysaccharide (LPS)‐induced pulmonary infection is one of the common causes of ALI.^5^ Therefore, further investigation into the underlying mechanism of LPS‐induced lung injury may contribute to a better understanding of ALI pathogenesis and facilitate the discovery of potential therapeutic targets.

Long noncoding RNAs (lncRNAs) are involved in various cellular mechanisms, ranging from gene regulation to protein translation and stability.^6^ LncRNAs have emerged as key players in the realm of inflammation and are believed to have significant involvement in disease progression.^7^ Furthermore, lncRNAs’ dysregulation has been observed in the lung tissues of LPS‐treated mice,^8^ suggesting a potential role for lncRNAs in the pathogenesis of ALI. The HOXA transcript at the distal tip (HOTTIP) is an lncRNA transcribed from the 5′ end of the HOXA gene locus.^9^ Previous studies have linked HOTTIP to the development of lung cancer and inflammatory associated diseases.^10^, ^11^, ^12^ Notably, HOTTIP knockdown has been shown to alleviate LPS‐induced myocardial apoptosis and inflammation.^13^ Giving these findings, we asked whether HOTTIP also contributes to the development of lung injury caused by LPS and sought to elucidate its function in LPS‐treated lung epithelial cells.

Surfactant protein C (SP‐C) is one of critical components of pulmonary surfactants, which are essential for maintaining lung defense and homeostasis.^14^ Evidence has indicated that SP‐C exerts a protective function in the context of lung injury.^15^, ^16^ SP‐C expression is frequently reduced in lung injury tissues, and this downregulation is associated with alveolar epithelial cell apoptosis and inflammation.^17^, ^18^ Moreover, research studies have shown that SP‐C interacted with LPS,^19^ a major contributor to lung injury, and displayed pro‐inflammatory properties.^1^ Notably, we found through the database prediction that among the four surfactants (SP‐A, SP‐B, SP‐C, and SP‐D), only the SP‐C promoter region has multiple CpG islands, suggesting that SP‐C may have DNA methylation. What is more, we predicted and found that HOTTIP and DNA methyltransferase‐1 protein (a key enzyme that maintains DNA methylation^20^) have the mutual binding possibility. Based on these findings, HOTTIP may affect SP‐C transcription by binding to DNA methyltransferase 1 (DNMT1).

Giving that HOTTIP and SP‐C have previously been implicated to be associated with inflammation and lung injury, here, we conducted a systematic experiment that aimed to explore the potential link between them and unravel the internal mechanism of these two molecules in lung injury caused by LPS. This work may offer valuable insights into potential therapeutic targets for the treatment of ALI.

## 2 MATERIALS AND METHODS

### 2.1 Cell culture

Primary human AEC II cells were obtained from Procell Co. Ltd. (CP‐H209). AEC II cells were cultured with DMEM/F12 (CORNING) and supplemented with 10% fetal bovine serum (FBS) and 1% penicillin/streptomycin, and the cells were maintained in a 5% CO_2_ atmosphere at 37°C. To induce a cellular model of LPS‐induced lung injury, AEC II cells were exposed to LPS at a concentration of 10 μg/mL for 24 h.

### 2.2 Plasmids’ construction and cell transfection

For gene interference studies, plasmids (si‐HOTTIP, si‐DNMT1, si‐SP‐C, oe‐HOTTIP, and their negative control) were purchased from Shanghai GenePharma Co. Ltd., and were transfected into cells using Lipofectamine^TM^ 3000 Transfection Reagent (L3000075, Invitrogen, Thermo). After a 1‐day incubation period, the transfection efficiency of plasmids was assessed using quantitative real‐time polymerase chain reaction (qRT‐PCR) and western blot analyses.

### 2.3 Quantitative real‐time polymerase chain reaction (qRT‐PCR)

HOTTIP, TNF‐*α*, SP‐C, IL‐1*β*, DNMT1, and IL‐6 expression levels were detected using qRT‐PCR analysis. In brief, the RNeasy Mini Kit (#74104, Qiagen) was utilized to extract total RNA and then the cDNA Transcription Kit (#4368813, Biosystems, Thermo) was used to remove genomic DNA contamination and reverse the RNA to cDNA. The PCR process was carried out using a real‐time PCR instrument. The expression of the target genes was normalized to GAPDH, and the relative expression levels were determined using the 2‐^ΔΔCT^ method. The primer sequences for the respective genes are provided in Table 1.

**TABLE 1:** List of primers used for quantitative real‐time polymerase chain reaction.

Genes	Forward (F)/Reverse (R)
HOTTIP	5′‐CCTAAAGCCACGCTTCTTTG‐3′
	3′‐TGCAGGCTGGAGATCCTACT‐5′
SP‐C	5′‐CACCTGAAACGCCTTCTTATCG‐3′
	3′‐TTTCTGGCTCATGTGGAGACC‐5′
DNMT1	5′‐ATGCTTACAACCGGGAAGTG‐3′
	3′‐TGAACGCTTAGCCTCTCCAT‐5′
TNF‐*α*	5′‐CCCCAGGGACCTCTCTCTAA‐3′
	3′‐TGAGGTACAGGCCCTCTGAT‐5′
IL‐1*β*	5′‐CGATGCACCTGTACGATCAC‐3′
	3′‐TCTTTCAACACGCAGGACAG‐5′
IL‐6	5′‐CCTTCCAAAGATGGCTGAAA‐3′
	3′‐ CAGGGGTGGTTATTGCATCT‐5′

### 2.4 Western blot

The levels of Bax, Bcl‐2, SP‐C, and DNMT1 proteins were determined using western blot assay. To extract total protein, RIPA buffer (#R0278, Sigma–Aldrich) was utilized to lyse the cells and extract total protein. The concentration of total protein was quantified using the BCA protein assay kit (Solarbio). Subsequently, the protein samples were subjected to SDS‐PAGE for separation and then transferred onto PVDF membranes. After blocking with skim milk, the PVDF membrane was incubated with the primary antibody overnight at 4°C, followed by incubating with the secondary antibody. An enhanced chemiluminescence reagent was used for color development, and the gray value of each band was analyzed using Image J software. The primary antibodies used in this study were as follows: anti‐Bax (ab32503, Abcam), anti‐Bcl‐2 (ab32124, Abcam), anti‐DNMT1 (ab188453, Abcam), anti‐SP‐C (32160702, Sigma–Aldrich), and anti‐GAPDH (ab9485, Abcam). GAPDH was chosen as the internal control for normalizing the analysis of target protein expression changes.

### 2.5 Fluorescence in situ hybridization (FISH) analysis

HOTTIP expression in AEC II cells was captured utilizing the HOTTIP specific probes, which were purchased from RiboBio (Guangzhou, China). Firstly, the AEC II cells were immobilized using 4% paraformaldehyde solution and made permeable by treating them with 0.5% Triton X‐100. Then the fluorescence in situ hybridization assay was performed according to the fluorescent in situ hybridization kit (Bes1001, BersinBio) guidelines. The nuclei were stained by DAPI. Subsequently, HOTTIP signal was detected with confocal microscopy (LSM800, Carl Zeiss).

### 2.6 Cell viability assay

The AEC II cells were seeded in 96‐well plates at a density of 1 × 10^4^ cells/well and incubated at 37°C, 5% CO_2_ for 48 h. A volume of 10 μL of the CCK‐8 reagent was introduced into each well, followed by an incubation period of 4 h. Then, DMSO (150 μL/well) was added and oscillated for 10 min. The assessment of cell viability was performed by measuring the absorbance at 450 nm using a microplate reader.

## 3 TUNEL

To detect the cell apoptosis, the AEC II cells were subjected to fixation and permeabilization, followed by incubation with a TUNEL reaction mixture containing TUNEL enzyme (11767305001, Roche, Basel, Switzerland) and TUNEL label mix (11767291910, Roche) at a temperature of 37°C for 1 h. Following rinsing with PBS, the cells were stained with 4′,6‐diamidino‐2‐phenylindole (DAPI). Imaging was performed using a fluorescence microscope.

## 4 ELISA

The levels of inflammatory cytokines secreted by AEC II cells were determined using specific ELISA kits. The ELISA experiments were performed following the TNF‐*α* ELISA kit (# KHC3013, Thermo), IL‐1*β* ELISA kit (ab214025, Abcam), and IL‐6 ELISA kit (ab178013, Abcam) instructions from the manufacturer.

### 4.1 Methylation‐specific PCR (MSP)

Methylation‐specific PCR (MSP) was conducted to assess the methylation status of the SP‐C promoter. Initially, genomic DNA of AEC II cells was isolated by Genomic DNA Purification Kit (K0512, Thermo), and the EpiJET Bisulfite Conversion Kit (K1461, Thermo) was used for sodium bisulfite treatment of the genomic DNA. The PCR amplification was carried out using a real‐time PCR instrument. After the reaction, the reaction product was taken and detected by agarose gel electrophoresis. Four pairs of primers were applied for the methylated and unmethylated targeted sequences to perform MSP. Primer sequences (designed using the MethPrimer design tool) are shown in Table 2.

**TABLE 2:** List of primers used in methylation‐specific PCR.

Gene		Forward (F)/Reverse (R)
SP‐C	Methylated	5′‐GATTTGTAGTATGAGGTAGAAGTCGA‐3′
		3′‐GATAACAAAAAAATACCAACCCG‐5′
	Unmethylated	5′‐TTGATTTGTAGTATGAGGTAGAAGTTGA‐3′
		3′‐CAATAACAAAAAAATACCAACCCAC‐5′

### 4.2 RNA immunoprecipitation (RIP)

si‐NC‐ or si‐HOTTIP‐ transfected AEC II cells were lysed in RIPA lysis and extraction buffer (#89900, Thermo). The extracted protein was subjected to an overnight incubation at 4°C with anti‐DNMT1 antibody (#PA5‐30581, Invitrogen) or anti‐IgG antibody (ab172730, Abcam). Subsequently, protein A/G Sepharose^®^ (ab193262, Abcam) was added and allowed to bind to the antibody–protein complexes for 1 h. Then, RNA was extracted using the Trizol reagent (#15596026, Invitrogen) to purify the RNA samples. Subsequently, qRT‐PCR was carried out to determine the RNA yield. The specific primers used for qRT‐PCR can be found in Table 1.

### 4.3 Chromatin immunoprecipitation (ChIP)

AEC II cells were cross‐linked with 1% formaldehyde, and then were collected and suspended in cold lysis buffer. Next, nuclei were collected to obtain DNA fragments of 200–50 base pairs. Magnetic beads with anti‐DNMT1 antibody (#PA5‐30581, Invitrogen) or anti‐IgG antibody (ab172730, Abcam) were then added to the lysate and subjected to an overnight incubation. After that, protein–DNA complexes were eluted from the beads, and the DNA products were amplified and electrophoresed on a 2% agarose gel.

### 4.4 Dual‐luciferase reporter assay

The wild‐type or mutant promoter region of SP‐C containing binding sites with DNMT1 were cloned onto the pGL3‐basic vectors to construct the luciferase reporter vectors. Then AEC II cells were inoculated into 48‐well plates (3 × 10^4^ cells per well). AEC II cells were cotransfected with negative control vectors or si‐DNMT1 by Lipofectamine 2000 (#11668019, Invitrogen) and incubated for a period of 48 h. After that, the luciferase activity was assessed utilizing the dual‐luciferase reporter assay system.

### 4.5 Statistical analyses

All experiments were conducted with a minimum of three independent biological replicates. The data were presented as mean ± standard deviation (SD). Statistical analysis was performed using GraphPad Prism 9.4.0 software. Student's *t*‐test and one‐way ANOVA, followed by Tukey's test, were employed for determining the statistical significance. A *p*‐value less than 0.05 (*p* < 0.05) was considered statistically significant.

## 5 RESULTS

### 5.1 HOTTIP knockdown inhibited LPS‐induced AEC II cell apoptosis and inflammation

**FIGURE 1:**
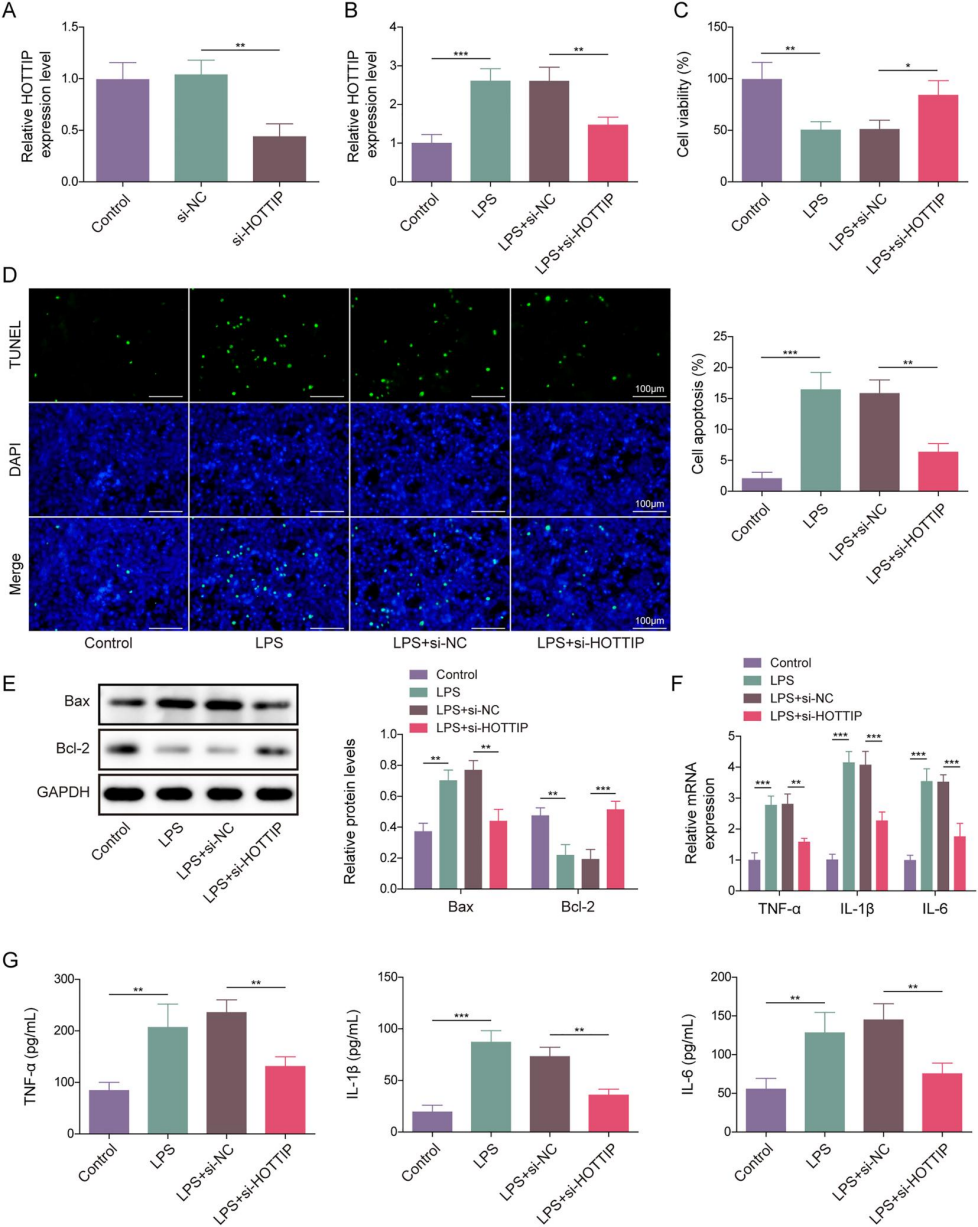
HOTTIP knockdown inhibited LPS‐induced AEC II cell apoptosis and inflammation. (A) The expression of HOTTIP in HOTTIP knockdown AEC II cells was detected by qRT‐PCR. (B) The expression of HOTTIP in LPS‐treated AEC II cells was detected using qRT‐PCR. (C) CCK‐8 assay was performed to examine the cell viability of AEC II cells. (D) TUNEL staining was used to detect the apoptosis of AEC II cells. (E) Western blot was performed to detect the expression of Bax and Bcl‐2 proteins in AEC II cells. (F) qRT‐PCR and (G) ELISA were performed to evaluate the level of TNF‐*α*, IL‐1*β*, and IL‐6 in AEC II cells. *p < 0.05, **p < 0.01, and ***p < 0.001.

To shed light on the function of HOTTIP in lung epithelial cell injury induced by LPS, we first constructed a specialized HOTTIP knockdown lung epithelial cell line in AEC II cells. As shown in Figure 1A, si‐HOTTIP markedly decreased the level of HOTTIP in AEC II cells. Subsequently, LPS, a potent inflammatory agent, was added to the cell line to stimulate apoptosis and inflammation in AEC II cells. As presented in Figure 1B, the administration of LPS notably upregulated HOTTIP expression in AEC II cells, and HOTTIP expression was downregulated after transfection with si‐HOTTIP in LPS‐induced cells, suggesting that HOTTIP might be involved in LPS‐induced lung epithelial cell injury. Furthermore, our observations revealed that LPS treatment resulted in a reduction in cell viability, an elevation in cell apoptosis, an upregulation of apoptosis‐related protein Bax, and a decrease in Bcl‐2, while HOTTIP knockdown reversed LPS‐induced cell death (Figure 1C–E). Moreover, the exposure to LPS led to a significant augmentation in both expression and release of TNF‐*α*, IL‐1*β* , and IL‐6, whereas HOTTIP knockdown abolished LPS‐induced cell inflammatory cytokines’ upregulation (Figure 1F–G). All the above studies indicate that HOTTIP knockdown inhibits LPS‐induced lung epithelial cell apoptosis and inflammation.

### 5.2 HOTTIP inhibited SP‐C expression by promoting the methylation of SP‐C promoter in AEC II cells

**FIGURE 2:**
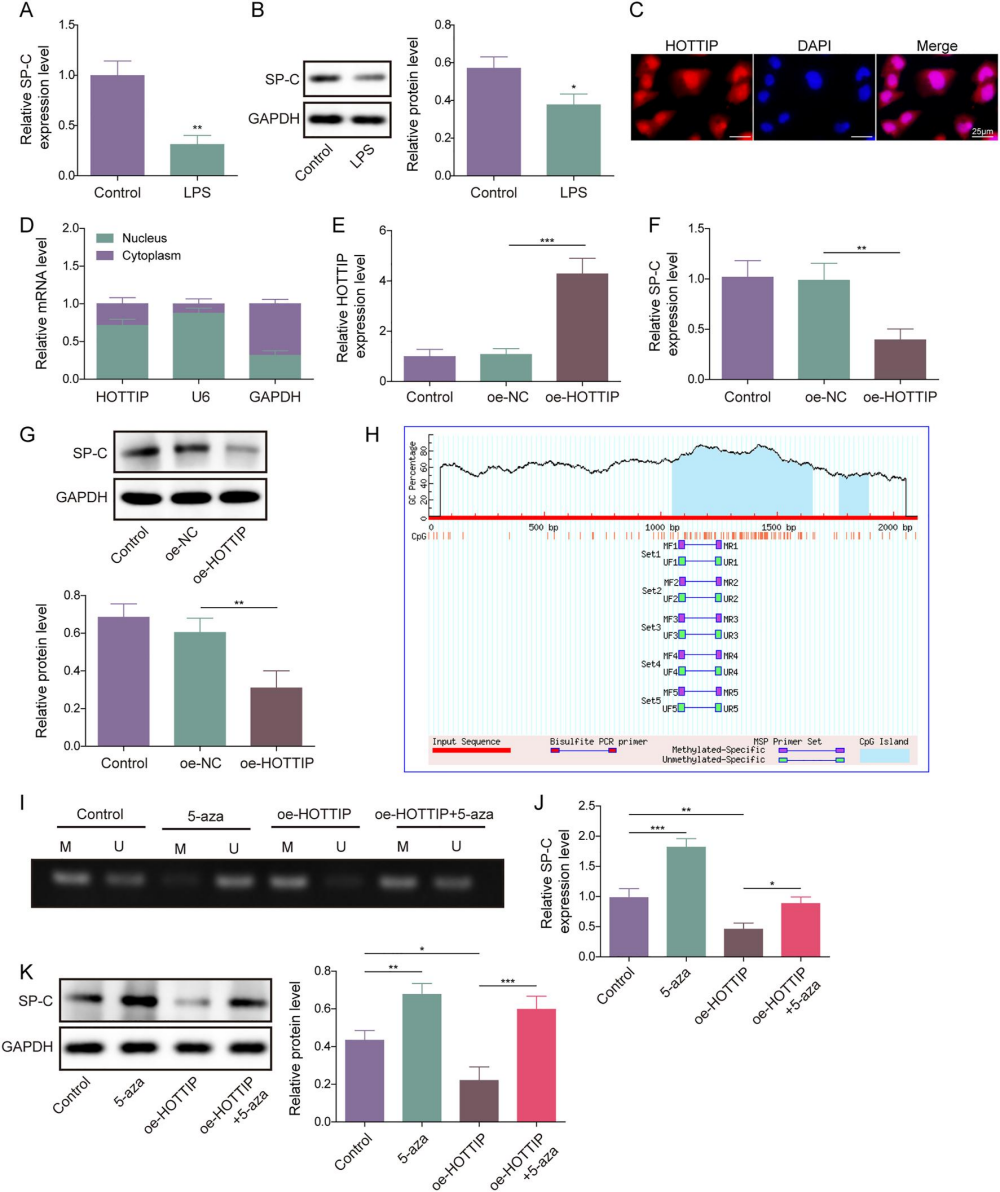
HOTTIP inhibited SP‐C expression by promoting the methylation of the SP‐C promoter in AEC II cells. (A) qRT‐PCR and (B) western blot were used to evaluate the mRNA and protein expression of SP‐C in LPS‐treated AEC II cells. (C) FISH was performed to detect the location of HOTTIP in AEC II cells. (D–E) qRT‐PCR was used to detect the mRNA expression of HOTTIP in AEC II cells and HOTTIP overexpression AEC II cells. (F) qRT‐PCR and (G) western blot were performed to detect the mRNA and protein expression of SP‐C in HOTTIP overexpression AEC II cells. (H) MethPrimer 2.0 (http://www.urogene.org/methprimer/) was used to predict the CpG island in the SP‐C promoter region. (I) MSP was performed to detect the methylation level of the SP‐C promoter. (J–K) qRT‐PCR and western blot were conducted to detect the mRNA and protein expression of SP‐C in AEC II cells with 5‐Aza‐CdR or/and oe‐HOTTIP. FISH, fluorescence in situ hybridization; MSP, Methylation‐specific PCR. *p < 0.05, **p < 0.01, and ***p < 0.001.

Prior investigations have provided evidence suggesting a strong correlation between the downregulation of SP‐C and the occurrence of apoptosis and inflammation in AEC II cells.^17^, ^21^ However, whether SP‐C regulates AEC II cell apoptosis and inflammation induced by LPS remains to be further investigated. Since HOTTIP is closely related to AEC II cell apoptosis and inflammation induced by LPS, we aimed to explore the possible links between HOTTIP and SP‐C in regulating these processes. Results showed that LPS significantly downregulated the level of SP‐C mRNA and protein (Figure 2A–B). Moreover, we observed that HOTTIP was predominantly localized in the nucleus (Figure 2C–D), indicating its involvement in gene expression regulation. Subsequently, as shown in Figure 2E, we generated an HOTTIP overexpression AEC II cell line and demonstrated that HOTTIP overexpression markedly reduced SP‐C mRNA and protein levels (Figure 2F–G).

To investigate how HOTTIP regulates SP‐C, we analyzed the gene information of SP‐C and discovered multiple high‐confidence CpG islands in its promoter region (Figure 2H). Further investigation indicated that the methylation of the SP‐C promoter region was significantly inhibited upon treatment with 5‐aza‐2′‐deoxycytidine (5‐Aza‐CdR), a potent inhibitor of methylation (Figure 2I), suggesting that the promoter region of SP‐C is susceptible to be methylated. Moreover, HOTTIP overexpression increased the methylation of the promoter region of SP‐C, which was reversed by 5‐Aza‐CdR (Figure 2I). Additionally, we found that 5‐Aza‐CdR significantly increased SP‐C mRNA and protein expression, whereas HOTTIP overexpression decreased SP‐C expression, and 5‐Aza‐CdR counteracted the influences of HOTTIP overexpression on SP‐C (Figure 2J–K). Based on these findings, it can be inferred that HOTTIP downregulates the expression of SP‐C at the transcriptional level by promoting the methylation of the SP‐C promoter region.

### 5.3 HOTTIP increased the methylation of SP‐C promoter via recruiting DNMT1 in AEC II cells

**FIGURE 3:**
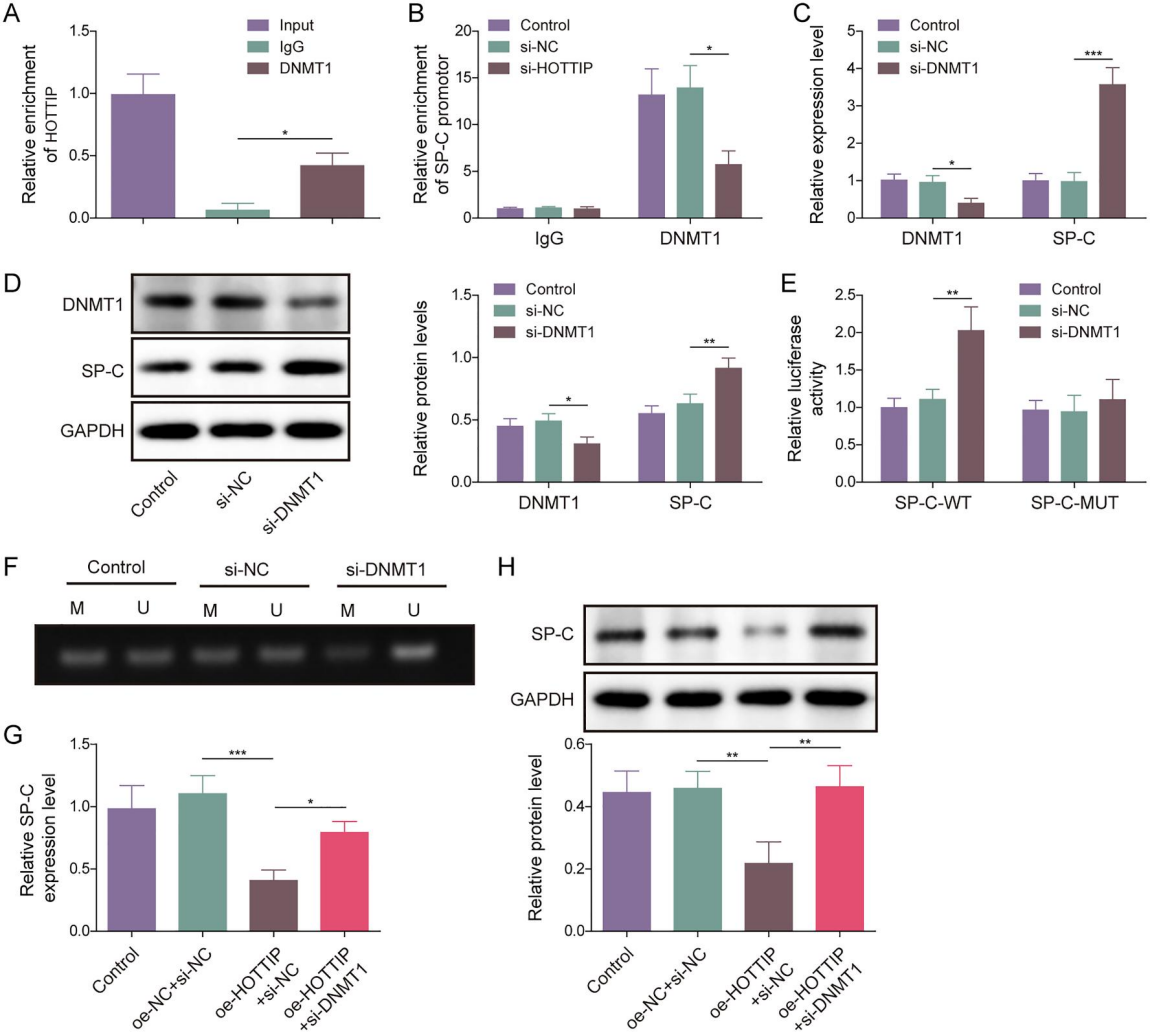
HOTTIP increased the methylation of the SP‐C promoter via recruiting DNMT1 in AEC II cells. (A) The binding interaction of HOTTIP and DNMT1 was examined by RIP assay. (B) ChIP assay was used to detect the enrichment of DNMT1 at the promoter region of SP‐C. (C) qRT‐PCR and (D) western blot were performed to detect the mRNA and protein expression of DNMT1 and SP‐C in AEC II cells. (E) Dual‐luciferase reporter assay was conducted to examine the binding ship of DNMT1 on the SP‐C promoter. (F) MSP was used to detect the methylation of the SP‐C promoter. (G) qRT‐PCR and (H) western blot were performed to detect the mRNA and protein expression of DNMT1 and SP‐C in HOTTIP overexpression or simultaneously DNMT1 knockdown AEC II cells. ChIP, chromatin immunoprecipitation; MSP, methylation‐specific PCR. *p < 0.05, **p < 0.01, and ***p < 0.001.

To elucidate how HOTTIP mediates the methylation of the SP‐C promoter, we turned attention to DNMT1, a crucial enzyme that adds methyl groups to the cytosine residues of CpG dinucleotides and maintains DNA methylation patterns.^22^ Hence, we investigated the function of DNMT1 in HOTTIP mediated DNA methylation of SP‐C. As shown in Figure 3A, HOTTIP was enriched by anti‐DNMT1, indicating that HOTTIP binds to DNMT1. Additionally, HOTTIP knockdown decreased the enrichment of DNMT1 on the SP‐C promoter (Figure 3B). Consequently, we hypothesized that HOTTIP might regulate the DNA methylation of SP‐C by recruiting DNMT1. To verify our hypothesis, we established a DNMT1 knockdown AEC II cell line and found that DNMT1 knockdown significantly downregulated DNMT1 and upregulated the mRNA and protein levels of SP‐C (Figure 3C–D). Moreover, the dual‐luciferase reporter assay showed that DNMT1 knockdown increased the luciferase activity of the SP‐C WT group but not the SP‐C MUT group (Figure 3E). A further study confirmed that DNMT1 knockdown reduced the methylation status of the SP‐C promoter (Figure 3F). Additionally, HOTTIP overexpression reduced mRNA and protein levels of SP‐C, while DNMT1 knockdown reversed these effects (Figure 3G–H). These results indicate that DNMT1 binds to the SP‐C promoter to promote SP‐C methylation and downregulate its expression. These data indicate that HOTTIP recruits DNMT1 to downregulate the SP‐C expression by promoting the methylation of the SP‐C promoter.

### 5.4 Inhibition of SP‐C reversed the effects of DNMT1 knockdown on LPS‐induced ACE II cell apoptosis and inflammation

**FIGURE 4:**
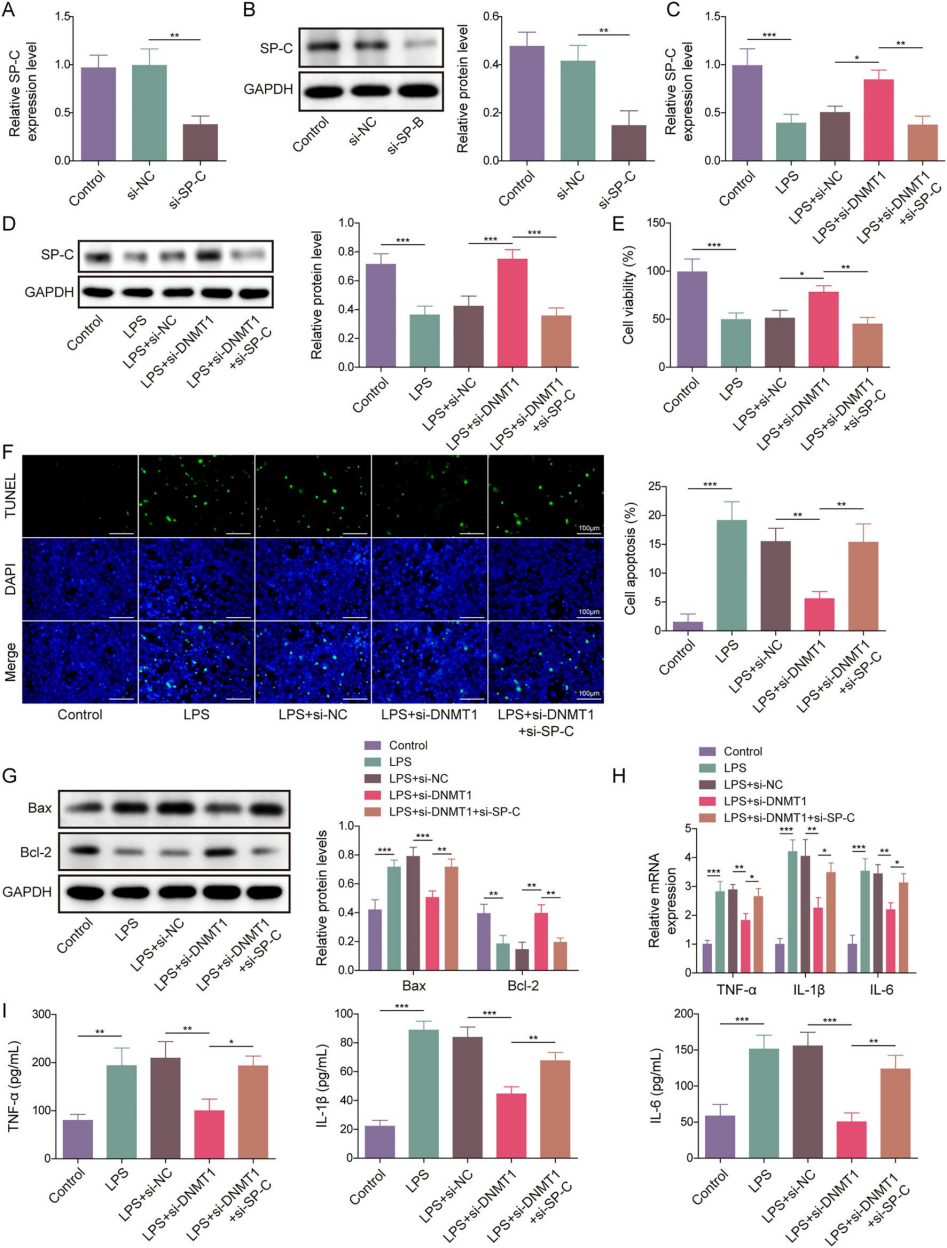
Inhibition of SP‐C reversed the effects of DNMT1 knockdown on LPS‐induced ACE II cell apoptosis and inflammation. (A) qRT‐PCR and (B) western blot were performed to detect the mRNA and protein expression of SP‐C in SP‐C knockdown AEC II cells. (C) qRT‐PCR and (D) western blot were conducted to detect the mRNA and protein expression of SP‐C in LPS‐treated SP‐C knockdown AEC II cells. (E) CCK‐8 assay was performed to examine the cell viability. (F) TUNEL staining was used to detect the apoptosis. (G) Western blot was performed to detect the expression of Bax and Bcl‐2 proteins. (H) qRT‐PCR and (I) ELISA were performed to evaluate the level of TNF‐*α*, IL‐1*β*, and IL‐6 in AEC II cells. *p < 0.05, **p < 0.01, and ***p < 0.001.

We further investigated whether DNMT1 affects the LPS‐induced lung injury by mediating SP‐C. As shown in Figure 4A–B, we established an SP‐C knockdown ACE II cell line. Then, we observed that LPS significantly downregulated the mRNA and protein levels of SP‐C, while DNMT1 knockdown counteracted the effect of LPS on SP‐C, and simultaneous knockdown of SP‐C and DNMT1 further reversed the effect of DNMT1 knockdown on LPS‐induced SP‐C expression changes (Figure 4C–D). Functional experiments revealed that LPS inhibited cell viability and promoted apoptosis, and DNMT1 knockdown rescued the effects of LPS by reversing the suppressed cell viability and the promoted apoptosis, while simultaneous knockdown of SP‐C and DNMT1 further reversed the ameliorative effects of DNMT1 knockdown on LPS‐induced inhibition of cell viability and increase in apoptosis (Figure 4E–G). Moreover, it was observed that LPS treatment led to an elevation in both the mRNA expression and secretion of TNF‐*α*, IL‐1*β*, and IL‐6, but DNMT1 knockdown counteracted the effects of LPS on those inflammatory factors. When SP‐C and DNMT1 were both knocked down, the effects of DNMT1 knockdown on LPS‐induced inflammation was nullified (Figure 4H–I). All these studies indicate that DNMT1 knockdown improves LPS‐induced ACE II cell apoptosis and inflammation via upregulating SP‐C expression.

### 5.5 Inhibition of SP‐C abolished the effects of HOTTIP knockdown on LPS‐induced ACE II cell apoptosis and inflammation

**FIGURE 5:**
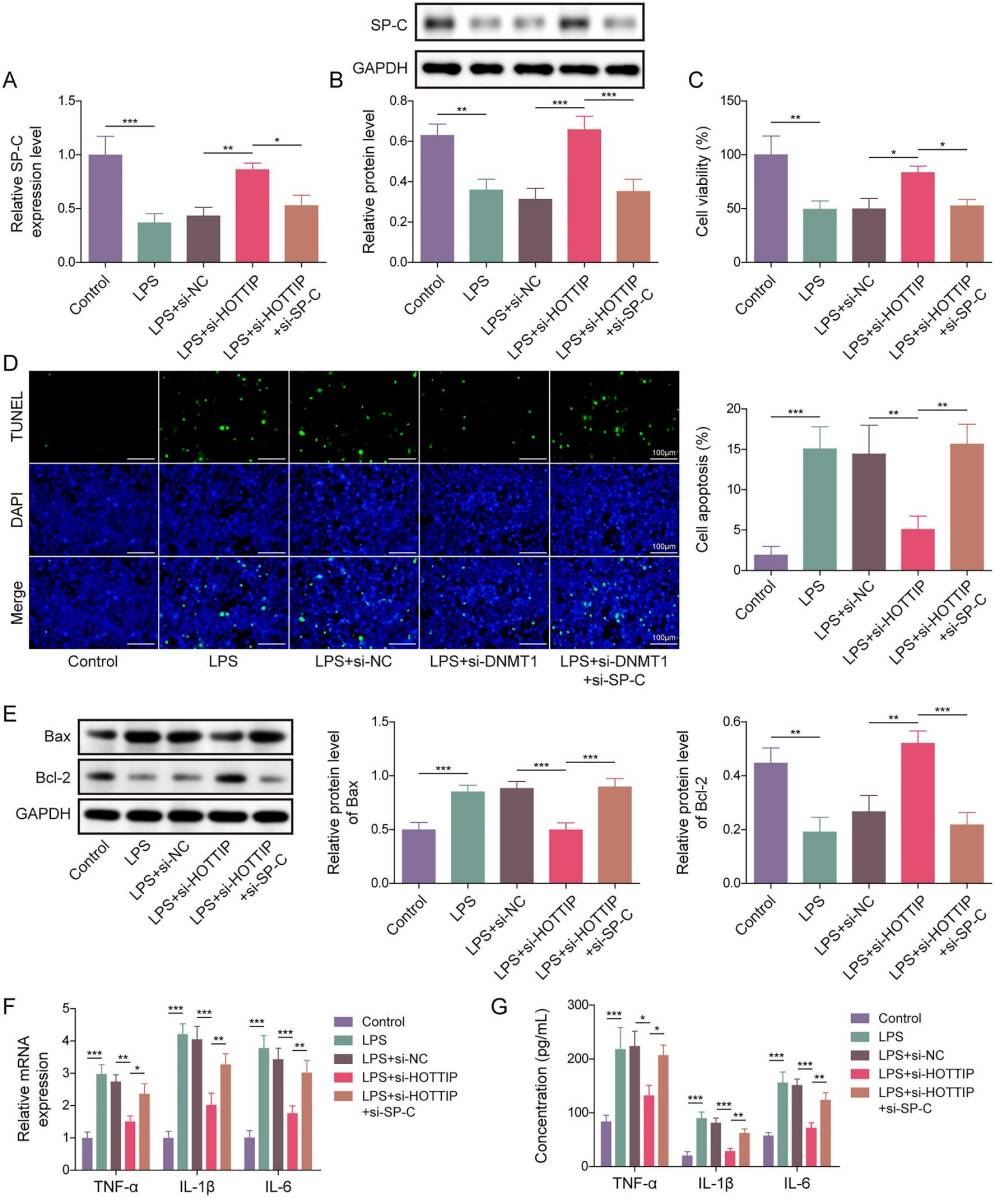
Inhibition of SP‐C abolished the effects of HOTTIP knockdown on LPS‐induced ACE II cell apoptosis and inflammation. (A) qRT‐PCR and (B) western blot were performed to detect the mRNA and protein expression of SP‐C. (C) CCK‐8 assay was performed to examine the cell viability. (D) TUNEL staining was used to detect the apoptosis. (E) Western blot was performed to detect the expression of Bax and Bcl‐2 proteins. (F) qRT‐PCR and (G) ELISA were performed to evaluate the level of TNF‐*α*, IL‐1*β*, and IL‐6 in AEC II cells. *p < 0.05, **p < 0.01, and ***p < 0.001.

Finally, we investigated whether HOTTIP regulates LPS‐induced lung cell injury by affecting SP‐C. Results showed that HOTTIP silencing reversed the inhibitory effect of LPS on SP‐C level, while simultaneous knockdown of both SP‐C and HOTTIP eliminated the effect of HOTTIP silencing on LPS‐induced changes in SP‐C expression (Figure 5A–B). Furthermore, functional studies revealed that HOTTIP knockdown inhibited LPS‐induced decrease in cell viability and increase in apoptosis, while further knockdown of SP‐C nullified the effect of HOTTIP knockdown on these processes (Figure 5C–E). Additionally, HOTTIP knockdown counteracted the influence of LPS on TNF‐*α*, IL‐1*β*, and IL‐6 production and release, while simultaneous knockdown of both SP‐C and HOTTIP negated the effect of HOTTIP knockdown on these inflammatory cytokines (Figure 5F–G). Overall, these findings suggest that HOTTIP promotes LPS‐induced ACE II cell apoptosis and inflammation by decreasing SP‐C expression.

## 6 DISCUSSION

The present study revealed that HOTTIP promoted LPS‐induced lung epithelial cell injury via the HOTTIP/DNMT1/SP‐C pathway. Mechanistically, HOTTIP recruited DNMT1 to the promoter region of SP‐C, where DNMT1 bound and promoted the methylation of SP‐C, leading to the inhibition of SP‐C expression, ultimately promoting LPS‐induced ACE II cell apoptosis and inflammation. Our study demonstrates that HOTTIP plays a key role in lung epithelial cell injury induced by LPS, possibly identifying a potential novel therapeutic target for ALI.

Previous studies have shown that HOTTIP was upregulated in fibrotic lung tissues and was related to cell proliferation and migration.^23^ Moreover, HOTTIP was upregulated in lung cancer tissues and promoted the tumor progression,^24^ suggesting that HOTTIP is crucial in maintaining lung homeostasis and that its dysregulation may contribute to the development of lung disease even lung cancer. Nevertheless, the function of HOTTIP in ALI remains unknown. Notably, a study by ZHU X J et al.^13^ reported that HOTTIP knockdown inhibited LPS‐induced myocardial apoptosis and inflammation, indicating that HOTTIP may play a role in regulating cell apoptosis and inflammation in various diseases. Here, we first reported that HOTTIP was dysregulated in LPS‐treated AEC II cells and promoted LPS‐induced AEC II cell apoptosis and inflammation. Our findings reveal that HOTTIP is an unfavorable factor in the pathogenesis of ALI and that targeting HOTTIP may be a promising strategy to prevent or treat ALI.

Studies have found that SP‐C was downregulated in different types of lung injury.^17^, ^25^ SP‐C is produced exclusively by AEC II cells and plays protective role in lung homeostasis.^26^ Previous studies have shown that the mice lacking the SP‐C gene exhibited increased susceptibility to LPS‐induced lung injury,^18^ and the loss of SP‐C in AEC II cells compromised their responses to infection.^21^ Herein, we found that HOTTIP promoted lung epithelial cell apoptosis and inflammation induced by LPS through inhibiting SP‐C. These findings suggest that SP‐C is a crucial factor in the pathway of LPS‐induced AEC II cell injury.

DNA methylation is a prominent epigenetic mechanism that influences gene activity, and the DNA methylation patterns of the CpG island usually led to gene silencing.^27^, ^28^ DNMT1 is a crucial enzyme that maintains the DNA methylation at CpG sites,^29^ which can interact with numerous genes and modulate their DNA methylation patterns. The DNA methylation pattern mediated by DNMT1 has been reported to be implicated in various diseases, especially cancers;^30^ it is considered as a potential therapeutic target in some cancer cases.^31^ It has been reported that DNMT1 downregulated miR‐124 to activate the nuclear translocation of protein pellino homolog 1 and interferon regulatory factor 5, thereby aggravating ALI in mice.^28^ Our finding revealed that there were high‐confidence CpG islands in the promoter region of SP‐C, and HOTTIP promoted the methylation of the SP‐C promoter, resulting in its inhibition. Additionally, we identified the critical role of DNMT1 in regulating SP‐C DNA methylation in lung epithelial cell apoptosis and inflammation induced by LPS, highlighting its significance in the pathogenesis of ALI. This study firstly reported the interaction among HOTTIP, DNMT1, and SP‐C in AEC II cells, indicating a network of regulatory mechanisms that may contribute to the pathogenesis of ALI. However, whether other DNA methyltransferases are involved in the regulation of SP‐C DNA methylation by HOTTIP deserves further investigation.

In a word, our study demonstrated that HOTTIP promoted LPS‐induced AEC II cell apoptosis and inflammation through epigenetically suppressing SP‐C. This study shed light on the mechanisms involved in the pathogenesis of lung epithelial cell injury and indicated that HOTTIP might be a potential prognostic biomarker in ALI. Based on these significant results, future studies should focus on designing in vivo experiments to confirm these findings in a living organism and assess the potential effectiveness of targeting HOTTIP in treating ALI.

## AUTHOR CONTRIBUTIONS

Shuang Li and Shuangjia Li contributed to the study conception and design. Material preparation, data collection, and analysis were carried out by Zhanqun Gao. Yang Liu wrote the initial draft of the manuscript, and all authors provided feedback and reviewed previous versions of the manuscript. The final manuscript was reviewed and approved by all authors.

## CONFLICT OF INTEREST STATEMENT

The authors declare no conflict of interest.

## ETHICS STATEMENT

Not applicable.

## CONSENT TO PARTICIPATE

Not applicable.

## CONSENT TO PUBLISH

Not applicable.

## Data Availability

Data sharing is not applicable to this article as no datasets were generated or analyzed during the current study.
