# Correction to “LncRNA HOTTIP promotes LPS‐induced lung epithelial cell injury by recruiting DNMT1 to epigenetically regulate SP‐C”

**DOI:** 10.1002/ccs3.12042

**Published:** 2024-10-31

Li, S., Li, S., Gao, Z. and Liu, Y. (2024), LncRNA HOTTIP promotes LPS‐induced lung epithelial cell injury by recruiting DNMT1 to epigenetically regulate SP‐C. *J. Cell Commun. Signal*, 18: e12020. https://doi.org/10.1002/ccs3.12020.

In the originally published article, the funding number was omitted. The correct sentence is:

The research was sponsored by the Scientific Research Project of Heilongjiang Health Commission (No. 2020‐332).

We apologize for this error.

